# Facile green synthesis route for new ecofriendly photo catalyst for degradation acid red 8 dye and nitrogen recovery

**DOI:** 10.1038/s41598-023-50930-7

**Published:** 2024-01-11

**Authors:** Nouf F. Al Harby, H. A. Fetouh, Mervette El-Batouti

**Affiliations:** 1 Department of Chemistry, College of Science, Qassim University, 51452 Buraidah, Saudi Arabia; 2 Chemistry Department, Faculty of Science, Alexandria University, Alexandria, 21526 Egypt

**Keywords:** Environmental chemistry, Pollution remediation, Nanoscale materials, Structural materials, Engineering, Materials science

## Abstract

This study novelty is that new photo catalyst prepared from sustainability low cost precursors. Dark red color hydrogel composites have been easily prepared from gelatin biopolymer using a simple sol–gel method. Gelatin doped by cobalt chloride, and silver nanoparticles (SNPs) in the presence of traces amount of sodium dodecyl sulfate surfactant and calcium chloride. Water-insoluble Gelatin composites are thermally stable photocatalysts for the degradation of toxic anionic acid red 8 dye. Promising photodynamic activity confirmed by fluorescence emission at *λ*_max_ 650 nm. Optical absorption in Vis. light enhanced photo catalytic activity. Silver nanoparticles enhanced crystallinity, and improved optical properties and porosity. Dopants by CoCl_2_ and silver nanoparticles increased band gap of gelatin composites from (1.82 to 1.95) indicating interfacial charge separation. Low band gaps improved photo catalytic activity. Optical band gaps (Eg) lower than 2.0 eV indicates high catalytic activity in the photo degradation acid red 8 dye using Vis. light, wavelength 650 nm. Percent removal efficiency (%Re) of the dye at 500 ppm initial concentration, pH 1, contact time 30 min., and 0.20 g L^−1^ dose photo catalyst reached 95%. pH not affects removal efficiency. So, gelatin composites removed AR8 dye by photodegradation mechanism rather than adsorption due to photodynamic activity. Kinetics of photodegradation followed pseudo first order kinetic with rate constant k_1_ 5.13 × 10^−2^ min.^−1^ Good electrical conductivity and magnetic properties (effective magnetic moment (*µ*_eff_ 4.11 B.M) improved dye degradation into simple inorganic species. Nutrients NH_4_^+^, and NO_3_^−^ degradation products recovered by using alumina silicate clay via a cation exchange mechanism.

## Introduction

Water is: the main resource for life on earth. Water challenges require efficient water treatment technologies Rapid grow global population and improved living standard continuously drive up water demand. Global climate change destabilizing water supply. Water contamination with dyes is a serious global environmental problem^1,2^.

Dyes widely applied in textiles, organo synthesis, paper pulping, anticorrosive protective coating, leathers and food. Seven thousands tons of synthetic dyes produced all over world. The textile industries which release the highest amount 54% of synthetic dyes in wastewater. Little amount of dye released in water decrease water quality and prevents both light and oxygen from reaching into water. Dyes accumulation in water possesses threat ecological system due to toxicity on human health and aquatic living organisms. Dyes are non-biodegradability, and resist photo degradation and thermal^3^. The most efficient adsobent for removal dyes are hydrogels.

Reported adsorbents for dye removal or pollutants destruction not included optically active hydrogel: Ecofriendly covalently immobilized enzyme nano composite destruction of direct Red 23 dye^1^; Nanoparticle and modelling of its photocatalytic dye degradation from colored wastewater^2^, ZnO nanoparticles photo catalyzed degradation Basic Blue 41 and Basic Red 46 dyes^3^; cadmium selenide quantum dot-zinc oxide composite is efficient photo catalyst for various dyes^3^; porous aminated PAN/PVDF composite nanofibers by electrospinning: Characterization and Direct Red 23 removal^4^, azo dyes removed by bio-nano composite whey protein nano fibrils and nano-clay^5^, Clay-based electrospun nano fibrous membranes for colored wastewater treatment^6^, metal–organic framework^7^. Aminated nanoporous nanofiber non selective biosorbent for dye^8^, (vinyl alcohol)-triethylenetetramine nanofiber by glutaraldehyde^9^, Graphene oxide nanosheet, Surface functionalized graphene oxide nanosheet for dye removal^10^, NiO‐MnO2 nanocomposite^11^, Silica nanoparticle for cationic dye^12^.

Hydrogel: soft porous 3D network cross-linked insoluble hydrophilic polymer swell and becomes rubbery soft in aqueous solution without splitting; synthesized from natural biodegradable nontoxic and cheap polysaccharides, gelatin, cellulose, denatured collagen alginate^13,14^. Excellent physical properties enhanced adsorption capacity^15^. Mechanical strength improved by crosslinking and incorporation of TiO_2_ nanoparticles NPs^16^.

Superabsorbent hydrogels have high swelling. Hydrogel is classified into natural hydrogels such as biofilm, gelatin, and sand sugar; synthetic hydrogel; homo, -co-polymers and multi interpenetrating polymer (IPN: hydrophobic, hydrophilic and ionic) hydrogel^17^ Hydrogel involves a network of two different cross-linked polymers, semi IPN: cross-linked-and non-cross-linked polymer; amorphous, semi crystalline, and crystalline; matrix, film, microsphere; neutral, ionic, amphoteric zwitter ionic)^18^. Physical crosslinking is favored over chemical crosslinking^18^; Rapid Gamma γ rays crosslinking improved mechanical strength^19^. Microwave rapidly induced grafting in water solvent (no initiator/catalyst used; water converts absorbed microwaves into heat energy). Time consuming conventional grafting requires an inert atmosphere, initiator/catalyst and involved competing homo polymerization^19^. Microwave grafting polysaccharides such as cellulose, starch, and guar gum (GG) improved mechanical, thermal properties^20^. Microwave grafting of guar gum composites by Na acrylamide increased grafting percentage^21,22^. Polyacrylamide is grafted by silica, aniline, and polyethylene glycol^23^. Swelling increased by increasing attractive forces among constituents of hydrogel^24^. Conventional hydrogel is heterogeneous, fragile with limited swelling and poor response to pH and temperature had little applications in water treatments^25^.

Functionalized hydrogel have high surface area to volume ratio (increased active sites; optical activity, Swelling enhance photo catalysis kinetics, water retention, bio compatibility, mechanical strength enhanced durability, recyclable. Disadvantages includes fragility, photo degradation on aging, limited shelf life: performance diminishes with time, requiring regeneration replacement.

Hydrogel composites are comparative to conventional photo catalysts, reusability, recovery from large volume systems can be challenging require additional steps. Reaction rate: Some hydrogel composites exhibit slower photo catalytic reaction rates compared to conventional solid-state photo catalysts due to diffusion limitations. Thermal sensitivity: Hydrogels sensitive to temperature fluctuations, which can affect their physical structure and, consequently, their photo catalytic efficiency. Complex Synthesis: Preparation hydrogel photo catalysts can involve complex synthesis protocols, which may hinder large-scale production and widespread application. While hydrogel composite photo catalysts show promising applications due to their customizable properties and effectiveness under light irradiation, challenges related to mechanical strength, longevity, and cost need to be addressed for their practical and widespread use in environmental and energy sectors.

The cobalt transition metal is a nontoxic inorganic metal of efficient catalytic activity^26,27^.

A hybrid organic–inorganic gelatin composite containing dual catalyst cobalt and AgNPs has not been reported yet. Anti-bacterial gallic acid-AgNPs hydrogel absorbs at *λ*_max_. 808 nm in near-infrared radiation^28^. Antibacterial AgNPs-polyvinyl alcohol used only in wound healing and dressing and tissue engineering^29^. Gelatin-alginate used in drug delivery had tunable physical properties, mechanical strength and swelling control^30^. Hydrogels containing AgNPs used in: wound healing^31^. AgNPs-chitosan-poly ethylene glycol (PEG) hydrogel used as an antioxidant; AgNPs-hydrogels applied in medicine: composites alginate/gelatin for wound healing; AgNPs-lignin hydrogel is antibacterial; PVA/SNPs hydrogel is conductive biosensor^32^. Only one hydrogel: carboxy methyl cellulose-gelatin AgNPs prepared by microwave method was used as an adsorbent for Congo-red and rhodamine B dyes^33^. No research articles reported about AgNPs hydrogels since 2020 (AgNPs Carbopol 940 gel), cobalt NPs-polymer hydrogel catalyzed reduction of nitro-compounds^34^.

AR8 dye is carcinogenic toxic dye and a serious pollutant in industrial (textile and tanneries) wastewater; harms the ecosystem, changes water quality and decreasing dissolved oxygen; difficult to remove during wastewater treatment^19^.

Photo catalyst composites containing TiO_2_ had large Eg 3.2 eV absorb at 387 nm in UV region of electromagnetic radiation. Compositing TiO_2_ with metal or non-metal slightly decreased Eg^35^. Non-toxic perovskite metal oxide semiconductors are heterogeneous photocatalysis that have low Eg but are expensive and unstable^36^. Lanthanum and toxic lead perovskites are efficient photocatalysts for the degradation methyl orange^37^. Photodynamic activity and photo excitation facilitate e-hopping mechanism^37^.

This study aims: Prepare new safe photo catalyst gelatin composites using low cost sustainable cheap precursors for degradation AR8 dye. Gelatin biopolymer had limited application due to uncontrolled hydration, viscosity changes on storage, to rapid biodegradation and contamination. Doping improve physicochemical and optical properties of gelatin hydrogel. This study: contributions knowledge advancement in photo catalysis, innovation design hydrogel composite novel photo catalysts by using natural gelatin doped by Co absorbs in Vis. light; providing new insights into photo catalytic degradation AR8 on hydrogel matrices. Using sustainable biodegradable non-toxic constituents align with green chemistry principles. Simple synthesis method could be repeated for scalability and commercialization Lab. scale synthesis. Economic Analysis: gelatin composite cheaper than conventional photo catalysts.

## Material and methods

Chemicals used in this study are high purity analytical grade reagents. All chemicals (Gelatin, Co(II) chloride.6H_2_O, AgNPs (zeta potential -28 mV), calcium chloride, poly methyl methacrylate (PMMA) resin, sodium dodecyl (SDS) surfactant, and AR8 dye) have been purchased from Sigma Aldrich Co.

Gelatin composites: molar ratio (gelatin_100-*x*_ CoCl_2*x*_), 0.001 M CaCl_2_ (gelling agent). The appropriate weights of these constituents dissolved in 50 mL double distilled water. AgNPs and SDS anionic surfactants are used for solubilizing water insoluble constituents. dopants were added, pH 8, agitation 50 rpm 2 h at 40 °C. Heating enabled sol–gel transition. Hydrogel formed on solved evaporation under vacuum^38^ Samples constituents of gelatin composites are represented in Table 1:

**Table 1:** Chemical composition and physical characteristics of the hydrogel.

Sample number	%Yield	Gelatin	CoCl_2_	CaCl_2_	AgNPs ppm	SDS	PMMA
S1	90.1	0.95	0.05	0.01	–	0.1	0.05
S2	86.1	0.90	0.10		5		
S3	85.2	0.85	0.15		10		
S4	93.0	0.80	0.20		20		

**Figure 1:**
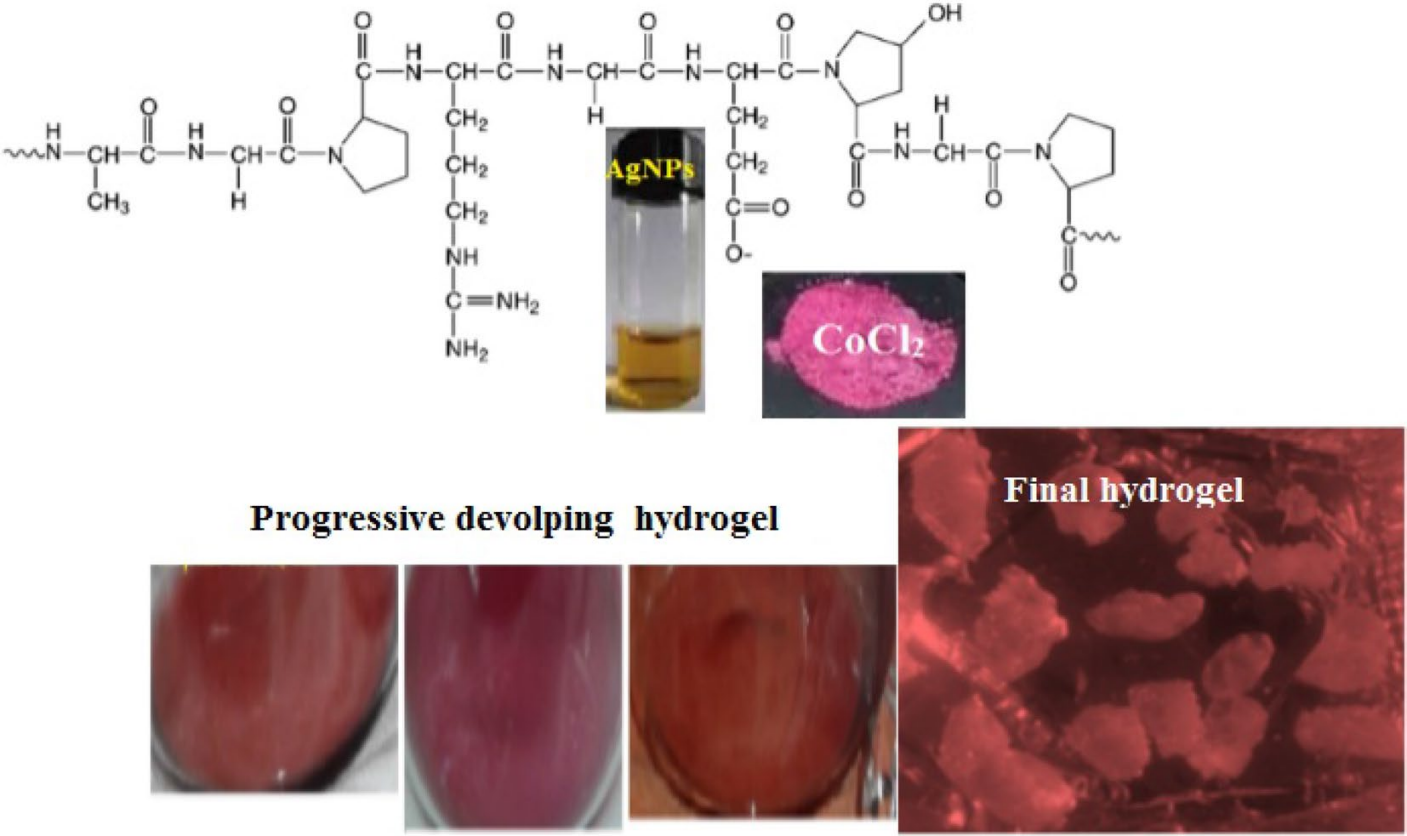
Visual inspection of network hydrogel gelatin doped cobalt chlorides, 20 ppm AgNPs.

Salts mixture is continuously agitated in double distilled water till attaining homogeneous saturated solution that was filtered and left covered with porous filter paper. After 24 h., dark red viscous hydrogel is obtained. The hydrogel lyophilized, freeze dried at 4 °C under vacuum gave dark red powder that annealed at 120 °C for 2 h^38,39^. Visual appearance in Fig. 1.

PMMA resin increased mechanical strength of hydrogel to tolerate physical stress and improved shelf life. 20 ppm AgNPs increased intensity of red color. Safe physical crosslinking assisted by heating–cooling cycle causing ionic interaction and intra molecular hydrogen bonding between gelatin chains leaving toxic non-biodegradable residues^40^. Gelatin composite will be biodegradable have desirable habitat and be thermally stable with neither no unreacted monomer^41^. Physicochemical characteristics of AR8 dye^13^ are represented as:

Intense red color due to chromophores S = O, azo N = N, and auxochromes: sulphonic and OH functional groups.

Gelatin composites have been characterized using different spectroscopic methods of analysis: Fourier transformer Infrared (FTIR) spectra, Bruker Tensor 27FTIR-spectrophotometer, Germany at frequency range 400–5000 cm^−1^; Surface morphology of gelatin composites and native gelatin were characterized using JEOL Scanning Electron Microscope (SEM), model JSM-6010 LV, Japan. Sample in form of films mounted on specimen stabs, coated with Pt thin film by ion sputtering electrode to increase conductivity and interaction with electron beam, introduced into equipment on sample holder. Accelerated voltage focusing, working distance, etc. were adjusted for every sample to get the best images^19^ Powder X-ray diffraction pXRD patterns recorded at 25 °C at diffraction, reflection angles (2-theta, 2*θ*°) 5°-80°, scan rate 1° min.^−1^ at 0.02° step using Cu-Kα x-ray radiation of wavelength (λ) 1.5418 Å, acceleration voltage 40 kV using Bruker D8 advance diffractometer. Intensity (arbitrary units) of reflected X-rays plotted versus incidence and reflection angles 2*θ*°; Thermal gravimetric analysis (TGA), differential thermal analysis (DTA) and differential scanning calorimetry (DSC) carried out using Shimadzu DTA/TGA-50, heating rate 10 °C min.^−1^, in de-aerated Pt cell to avoid air oxidation, flow rate 20 mL.min^−1^ at temperature range: 25–800 °C using SDT Q600 V20.9 Build 20 instrument; UV–Vis. electronic absorption spectra done using Lambda 4B Perkin Elmer spectrophotometer at λ 200–900 nm; emission spectra recorded using DS-11 series Spectrophotometer/Fluorometer, DeNovix Inc., λ_excitation_:190–840 nm, Detector 2048 element CCD, Photodiode, Resolution:1.5 nm (FWHM at Hg 253.7 nm).

Inductively coupled plasma/optical emission spectrometry (ICP/OES) used in determination metal ion concentration (ppm) leached from gelatin composite (sample digested in conc. HNO_3_, injected into instrument. Metal detected as free aerosol gaseous atom.

A batch photodegradation experiment was carried out at low pH 3 (to enhance removal efficiency), different dose composite ultra-sonicated for 15 min. to increase surface area. Different initial concentration (C_i_) of AR8 dye added with constant stirring at 50 rpm, adjusted at pH 3.0, and magnetic stirring continued for 30 min. to attain equilibrium adsorption of dye on the catalyst surface. At different time intervals, 5 mL suspensions were collected and centrifuged. For both samples in the dark and after light irradiation (100 W fluorescent Vis.-light lamps incident on dye solution contain gelatin composite. Residual dye concentration in dark and light determined by UV–Vis. spectroscopy at *λ*_max_. 508 nm using a calibration curve. Photo degradation followed in aqueous dye solution at pH 3, 0.20 g dose photo catalyst, illumination time 1.0 h. Residual dye concentration in terms of absorbance is converted into concentration using molar extension coefficient (slope of straight line equals, ε: 2*10^4^ L mol^−1^ cm^−1^ ^19^ obtained from application Beers Lambert law following least square method, correlation coefficient R^2^ 0.9984.

Degradation efficiency of AR8 dye equivalent to %Re^19^:

1 $${\text{Re }}\% \, = \, \left( {{\text{Co }}{-}{\text{ Ct}}} \right) \, /{\text{ Co }} \times { 1}00$$

where Co and Ct are initial and residual dye concentration respectively. Removal percentage (% Re) of AR8 by adsorption or photo catalysis at equilibrium calculated using relation^19^:

2 $$\% {\text{Re }} = \, \left( {{\text{Co }}{-}{\text{ Ct}}} \right) \, \times {\text{V}}/{\text{Co}} \times {\text{w}}$$

where ${q}_{e}$ is AR8 dye concentration at equilibrium (mg/g), and ${C}_{i}$ , ${C}_{e}\mathrm{ are}$ initial and equilibrium dye concentration (mg L^−1^), V is solution volume, (L) and W mass (g) of photo catalyst.

Degradation data are linear fitted to different kinetic models^42^:

Zero-order kinetic model:

3 $$Q_{t} = Q_{o + } K_{o} t$$

where Q_0_, Q_t_ are initial and released concentration respectively (almost, Q_0_ = 0) and K_0_ is zero order release constant, concentration.time^−1^.

Pseudo first order (1°) kinetic

4 $${\text{ln }}\left( {{\text{Co}} - {\text{Ct}}} \right)/{\text{Ct }} = {\text{k}}_{{1}} {\text{t}}$$

where k_1_ is the rate constant of adsorption, min.^−1^ and t is contact time in min.

Pseudo second-order (2°):

5 $$\frac{t}{{q_{t} }} = \frac{1}{{k_{2 } q_{e}^{2} }} + \frac{t}{{q_{e} }}$$

Water absorption measurements (swelling) are carried out in triplicates (N = 3 for reproducibility) according to ASTM standard D-570–98. Composite sample is immersed in distilled water at 23 ± 2 °C for different time intervals. Sample is taken out from water and all surfaces debris are removed using clean dry cloth and accurately weighed. Water absorption is determined by weighing samples at regular time intervals^12^:

6 $$\% {\text{Water absorption }} = \, \left[ {\left( {{\text{W2}} - {\text{ W1}}} \right)/{\text{W1}}} \right] {1}00$$

where W1, W2 are sample weight before and after soaking respectively.

### Ethical approval

There is no ethical issue in the manuscript. Authors approved consent on participation.

## Results and discussion

**Figure 2:**
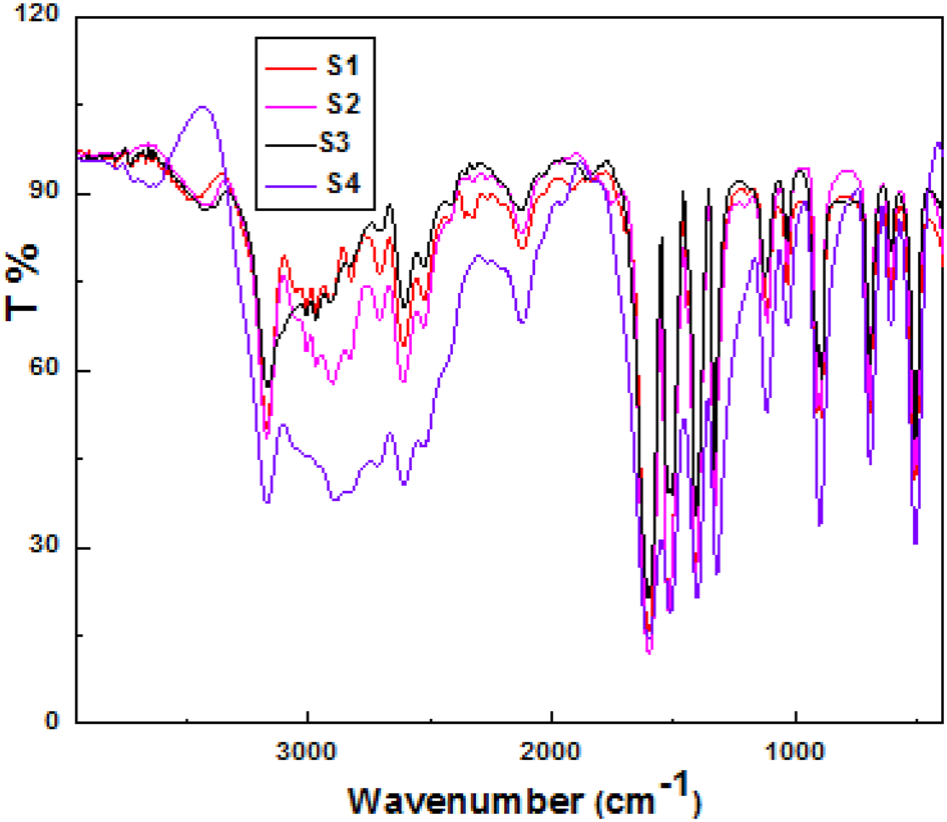
FTIR of hydrogel samples (**a**-**d**) samples 1–4 respectively.

Figure 2 shows FTIR spectra of gelatin composites:

Bands assigned to characteristic stretching frequency (wavenumber, cm^−1^), with increasing wt. % CoCl_2_, band intensity at 3600 cm^−1^–3000 cm^−1^ increased due to multiple OH groups. Vibration bands below 1000 cm^−1^ confirmed high force constant in Co-oxygen and Co-nitrogen bonds. Weak bands symmetric stretching NH, CH at 2812 cm^−1^. The band at 3034–3062 cm^−1^ became much weaker, blue shifted to 2733 cm^−1^ on binding through N, O. Bands at 649, 1041, and 1622 cm^−1^ due to stretch C–N, N–N, C = N bonds respectively slightly shifted, intensified as doping increased electron cloud. Bands at 1227, 1276, 1428 cm^−1^: C–N stretching, N–H deformation on binding CoCl_2_. Bands weakened and slightly shifted in position on doping. Intense band at 1378 cm^−1^ due to organic moiety of gelatin: AgNPs Bands at 520–537 cm^−1^ confirmed Co–N, Co–O bonds, and C-N. Peaks (NH_2_: symmetric, anti-symmetric NH and CN stretching. Intense band at 1380–1385 cm^−1^: C = C bond. Native gelatin retained in composites, peaks at 3460, 2920 to 2885, 1647, 1384, 1152, 1075, 1028, 557 cm^−1^ due to O–H, C-H, O–H, C-N, C-O(H), C–O–C stretching respectively. Co(II) ion binding gelatin via electron donors heteroatoms via N, O atoms. High delocalized electron density attained by dispersed AgNPs on gelatin polymeric matrix υ_C=N_, affected by interaction between Co(II) and π-electrons of C = O. Band at 1510–1608 cm^−1^due to stretching vibration C = N bonds. Coordination bond formed between ketonic C = O group of gelatin and Co(II) ion. Bonding includes Cl^−^ ion and coordinating water molecules. The most intense vibrations bands of sample confirmed strong bonding^43^.

**Figure 3:**
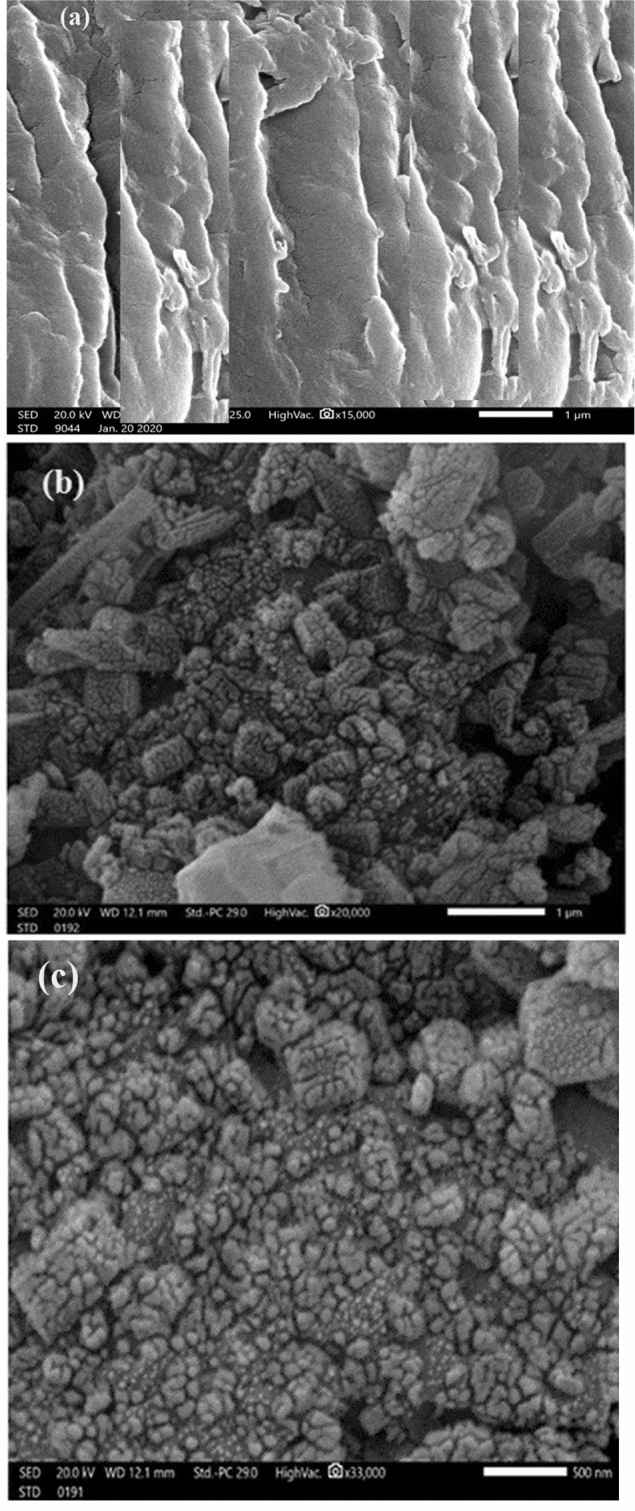
SEM micrographs: (**a**) gelatin, (**b**) sample 1 and (**c**) sample 4 respectively.

Figure 3 (a-c) showed SEM surface micrographs: surface morphology of gelatin composites showed (2D arranged chains at a small spacing distance) on binding Co(II) ions. Microstructure changed from semi crystalline and more appearance capsule shape into crystalline structure in the presence of AgNPs.

Gelatin exhibited continuous polymeric chains. This structure changed on doping by cobalt chloride and decoration by AgNPs. Smooth structure due to high cross-linking between polymer chains. N atoms of gelatin binding oxygen atom via intra molecular H.B. and Van der Waals interaction gave uniform small micrometer particle size^17^. 3D cross-linking increased surface area. PMMA chemically grafted hydrophilic functional groups of gelatins. Doping and grafting gelatin improved its polymer shell giving network hydrogel. NH, C = O, OH increases water entrapment into capillary porous.

Gelatin properties modified on doping. Sample 4 showed the most intense vibrational bands in FTIR spectra, so it was selected for further investigation. SEM images show changes in surface morphology of gelatin composites compared to native gelatin indicating grafting of dopants CoCl_2_ and AgNPs to backbone chains of gelatin.

**Figure 4:**
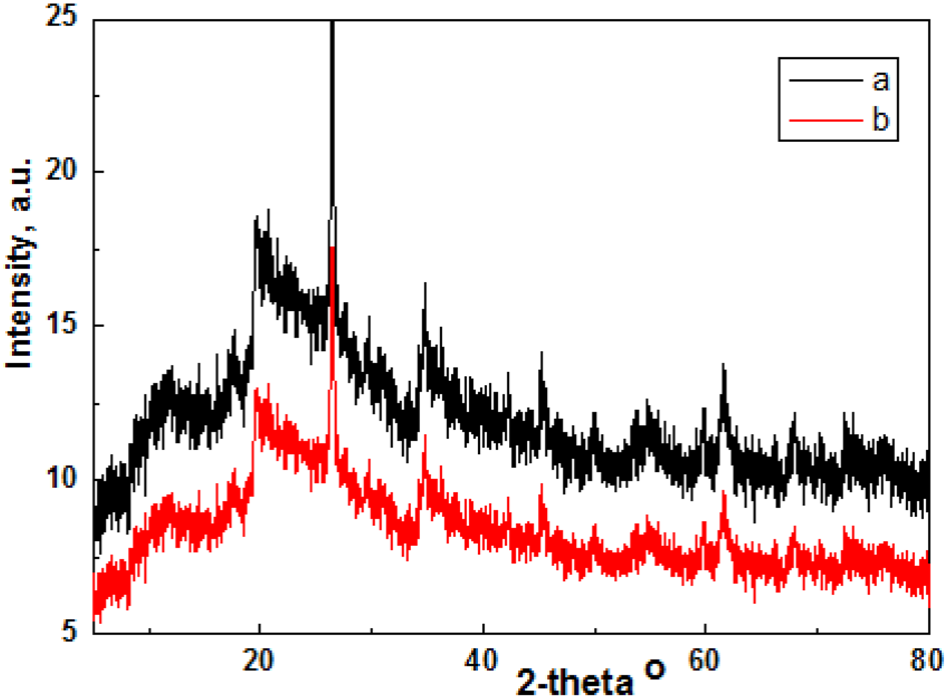
pXRD pattern of hydrogel: (**a**) S1 and S4 respectively.

pXRD patterns, Fig. 4 showed pure single phase semi-crystalline gelatin composites have crystalline and amorphous domains with increased surface area. Gelatin was responsible for the formation of a transparent polymer film. pXRD diffraction pattern revealed the formation of gelatin composites containing CoCl_2_ and AgNPs with characteristic diffraction peaks at 2*θ* 24°, 23° and 38°, 47°, 64° respectively^13–15^.

Amorphous composites improved catalytic activity.

**Figure 5:**
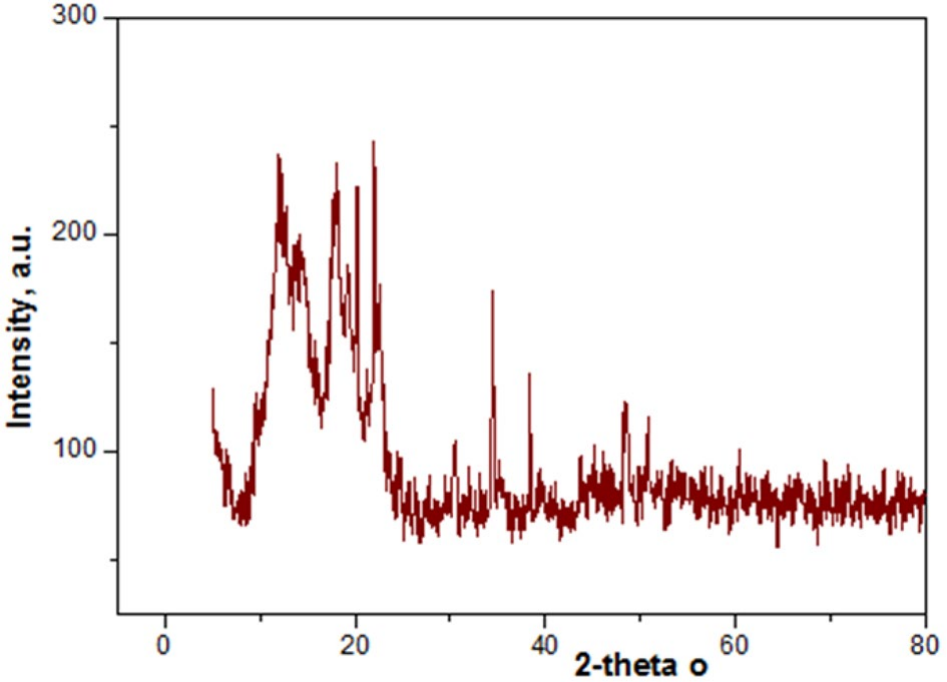
pXRD pattern of hydrogel S4 after 5 recycle reusability.

Figure 5 showed pXRD pattern foe AR8 dye after 5th repeated cycles reusibility.

Retained pXRD pattern of hydrogel confirmed reusability for many repetitions due to: Chemical Stability (unchanged chemically after degradation organic pollutants).

Mechanical stability. Hydrogels must maintain their mechanical integrity throughout the swelling and de-swelling by aqueous solutions during photo catalysis and photo stability against photo degradation.

**Figure 6:**
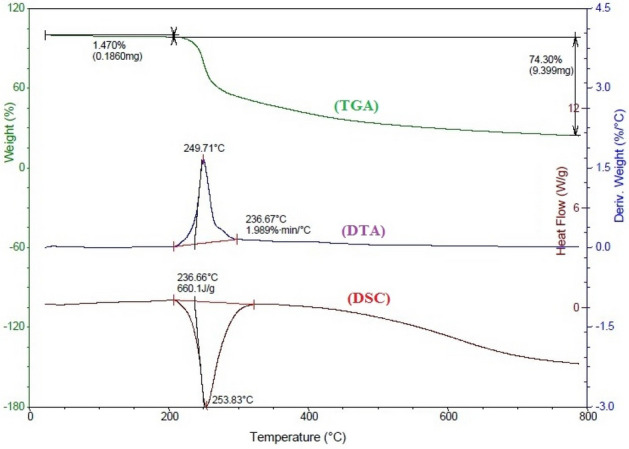
TGA, DTA and DSC thermograms of gelatin composite sample 4.

Thermal stability of gelatin composite sample4 is confirmed in Fig. 6. In TGA. Curves, weight loss: below 200 °C due to dehydration; above 200 °C for gelatin units’ degradation accompanied with phase change^15,19^.

Thermal behavior confirmed doping gelatin by cobalt chloride and AgNPs. TGA curves showed slight weight loss due to dehydration and loss both lattice and coordinated water molecules. DTA curves showed high Tm 240.71 °C confirmed thermal stability. In DSC, sample and reference sample have same or different masses and are kept at the same temperature. Energy given or removed from sample to maintain ΔT_sample-reference_, equal zero. The power energy change in the sample measured as a function of heat flow. Thermal transition changes specific heat and alters the power signal. Exothermic or endothermic peaks areas proportional to ΔH. Heat capacity C (heat Q absorbed by closed system of constant composition (dV = dN = 0) on heating 1 K^44^:

7 $${\text{C }} = {\text{ Q }}/{\text{DT}}$$

Heat capacity at constant pressure *C*_P_ differs than C_V_ because Cv depends on internal energy and work done on volume expansion. For solids at low temperatures *C*_P_ ≈ C_V_, when two pans heated, heat absorbed by the sample-temperature plot represent DSC curves. Heat flow: (heat, *q* supplied per unit time, *t*. Heating rate is temperature increase per unit time, *t*.

8 $$CP = \frac{heat \, flow}{{heating\, rate}} = \frac{\frac{q}{t}}{{\frac{\Delta T}{t}}} = \frac{q}{\Delta T}$$

**Figure 7:**
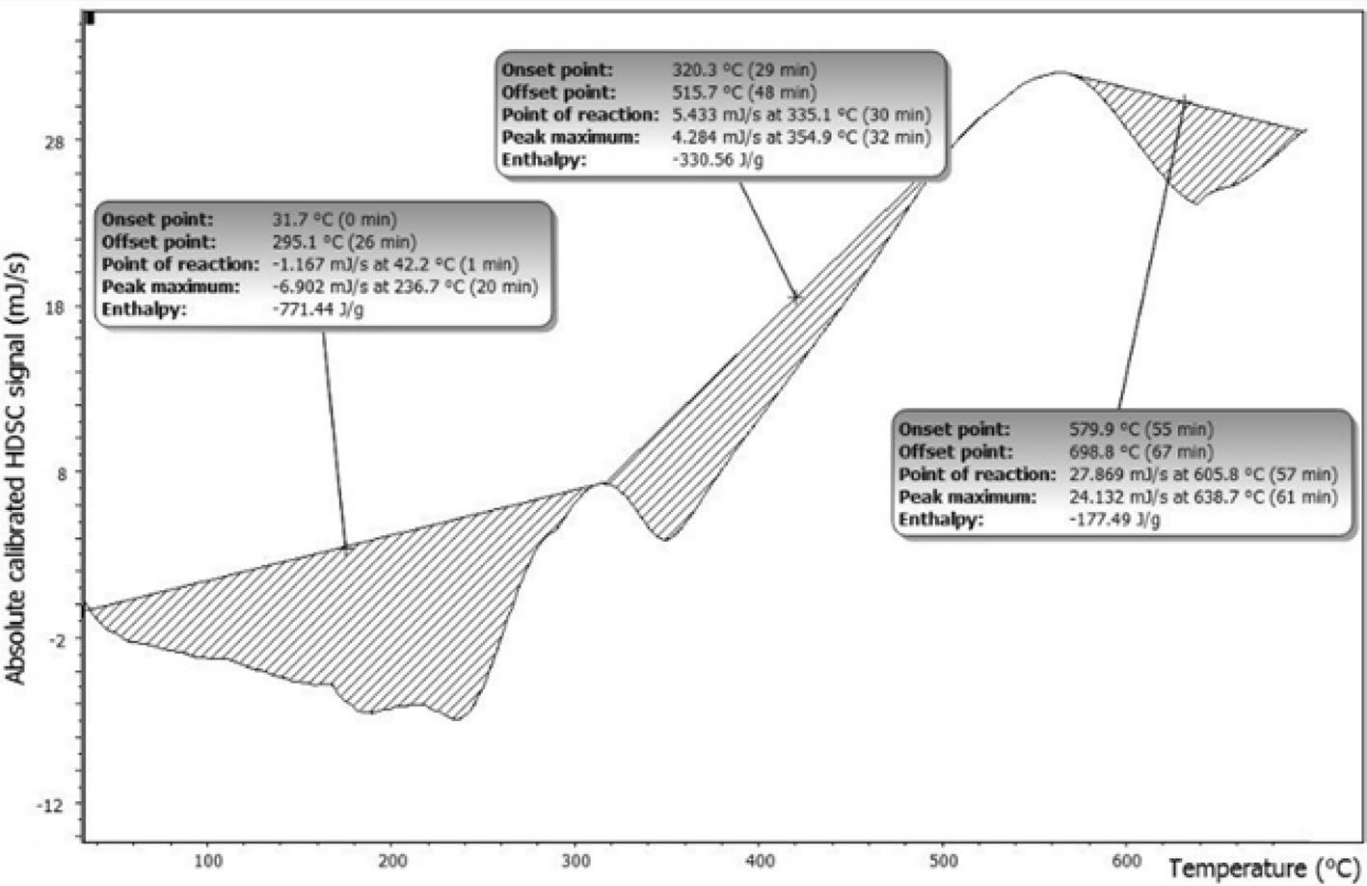
DSC thermal transitions: glass temperature Tg, crystallization, and melting.

DSC represented in Fig. 7 explores thermal transitions: glass temperature Tg, crystallization, and melting. Above Tg: *C*_P_ of sample increased and measured at the middle of the incline. *T*_crystallization_: above *T* g, mobility improved as kinetic energy increased atomic motion in ordered crystalline arrangements release heat.

The area under the crystallization peak gave exothermic ΔH _crystallization_ transition). Endothermic melting above *T*_c_ at *T*_melting_ that remains constant until complete melting, Area under peak equals latent heats of melting ΔH _melting_. Glassy and crystallization transition involves no peaks confirming the thermal stability of the gelatin composite, Table 2.

9 $${\text{C}}_{{\text{p}}} = {\text{ a T }} + {\text{ b }} = \, \alpha {\text{T}}^{{3}} + \, \gamma {\text{ T}}$$

**Table 2:** Thermal transitions and DSC parameters.

Sample	Thermal transitions (°C )	*C*_P_ (mJ)		
	T_g_	*T*_c_	*T*_m_	
S1	243	345	655	0.29
S2	113	328	486	0.36
S3	98	214	498	0.35
S4	265	344	560	0.50

Above 100 °C, almost all sample showed glassy transition followed by crystallization and melting^32^. Small *C*_P_ indicated the weak thermal conductivity of the samples confirming uses reduce thermal effect exothermic photo degradation.

The hydrogel composite withstand waste heat from exothermic reactions occur during photo catalysis without significant thermal degradation.

The main factors: pH, temperature, dose, contact time, and initial dye concentration affecting experimental %Re of AR8 dye in dark and light identified from Plackett–Burman statistical analysis. Five independent variables were screened in six combinations, each variable organized, high ( +) and ( −) level. Averages %Re of duplicates determinations taken as responses^45^:

10 $${\text{Factor observed effect }}\% {\text{R }} = \overline{\% R}_{ + 1} - \overline{\% R}_{ - 1}$$

${\stackrel{-}{\%R}}_{+1}$, ${\stackrel{-}{\%R}}_{-1}$ are high and low setting respectively.

11 $${\text{Main average variable effect }} = \, \left( {\sum \% {\text{R}} + \, - \sum \, \% {\text{R}} - } \right)/{\text{number of trials }}\left( {\text{N}} \right))$$

% R_0_._0_ means no effect. Positive and negative main effects indicate variables near or apart from optimum respectively, Table 3. Control factors with effects magnitude qualified and statistically significant effects determined. Optimal conditions determined by combining levels of factors had the highest main effect. Student's *t*-test explored the statistical significance of regression coefficients of variables^45^.

**Table 3:** Plackett–Burman matrix and levels of independent variables affect %Re.

Trials	pH	Contact time	*t*°C	Dose	Agitation	%R
1	1	− 1	− 1	1	− 1	2.69
2	1	1	− 1	− 1	1	0.75
3	1	1	1	− 1	− 1	2.34
4	− 1	1	1	1	− 1	0
5	1	− 1	1	1	1	1.15
6	− 1	1	− 1	1	1	2.73

Parameters enhanced %Re are dose initial concentration and time. Temperature and pH were not affected. Strong electrostatic interaction on the surface of positively charged composite in solution below its pH of zero PZC (5.3) did not alter %Re indicated that gelatin composites act by photocatalysis, not adsorption. The surface charge of gelatin composite at different pH did not affect %Re. %Re was independent of temperature. Statistical parameters optimized in Table 4^25^.

**Table 4:** Statistical analysis of Plackett- Burman method.

Variable	Coefficients	SD.E	*t*- Stat	*P*	Main effect
pH	− 0.27	0.22	− 1.20	0.44	Insignificant
t(min.)	0.13	0.22	0.58	0.67	–
Light intensity	− 0.36	0.22	− 1.60	0.036	Significant
Dose	− 0.55	0.22	− 2.44	0.02	–

In light, both Ci and light intensity control %R.

**Figure 8:**
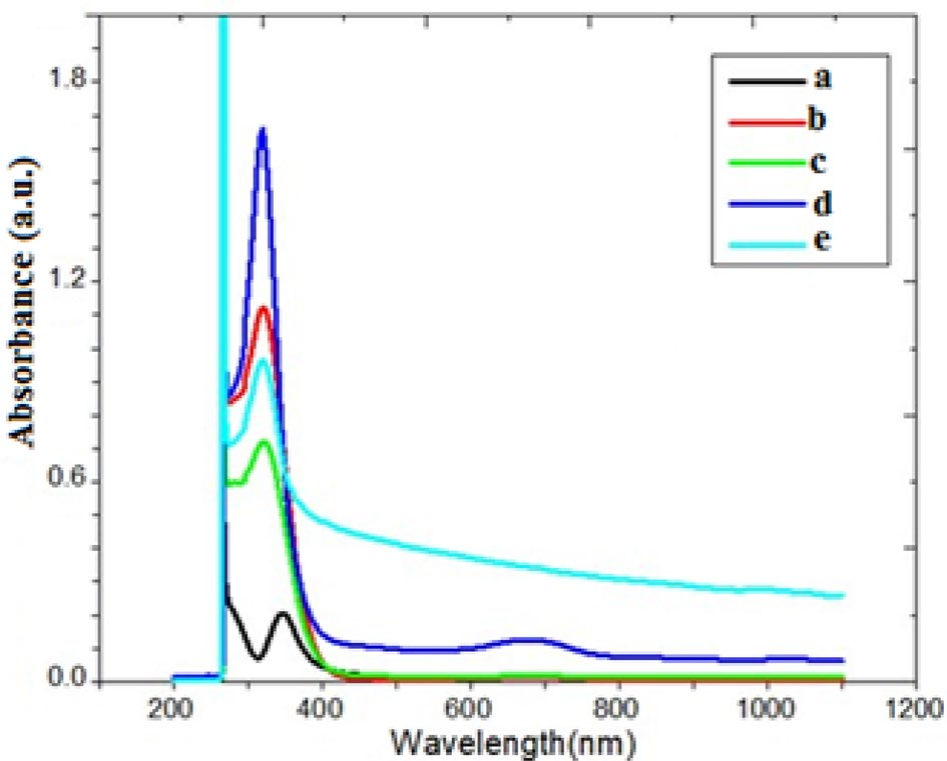
UV–Vis. absorption spectra: a) gelatin and gelatin composites S1-S4 respectively.

The Fig. 8 UV–Vis. absorption spectra of native gelatin and gelatin composites due to electronic transitions from the ground state into the excited state.

Sample 4 showed three absorption peaks at 262, 314 and 715 nm. Weak absorption at 715 nm is due to intra-molecular charge transfer. The spectral redshift of sample 4 (curve e) improved photo catalytic activity. Intensity of UV–Vis. absorbance bands depend on wt.% Co(II) ion for same chromophores^42^.

12 $${\text{ fraction absorption }}\alpha = \frac{2.3042 \times absorbance A}{{{\text{sample thickness}} t}}$$

**Figure 9:**
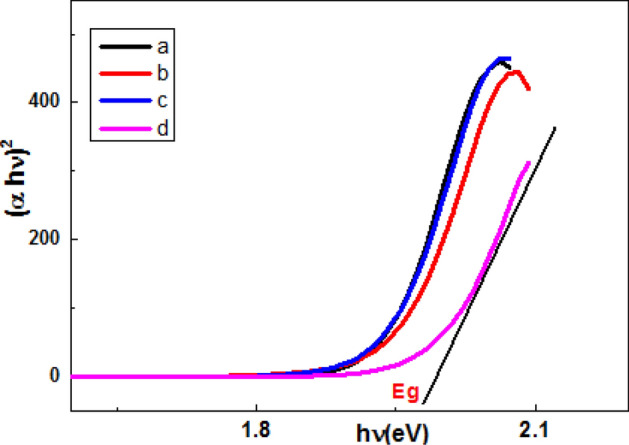
Tauc’s plots calculation band gap (**a**-**d**) for samples (1–4) respectively.

Figure 9 showed concentration dependence of band gap Eg that controlled UV-absorption coefficient depends on photon energy $h\upsilon$ :

13 $$\alpha h\nu = A\left( {h\upsilon - {\text{Eg }}} \right)^{r} \,{\text{ or }}\,\left( {\alpha {\text{h}}\nu } \right)^{{2}} = {\text{ A}}\left( {{\text{h}}\nu - {\text{Eg}}} \right)$$

where *ν* frequency of incident radiation inversely proportional to wavelength (*λ*); A: constant depend on reduced masses of electron–hole pair and refractive index of the composite material and exponent *r* depends on nature of electronic transition; *r* = 2, ½ for indirect transition and for allowed direct transition respectively^42^.

Doping by CoCl_2_ and AgNPs increased band gap of gelatin composites from (1.82 to 1.95) due to separation of charge (electron–hole) charge carries. All composite samples have suitable low Eg to be used as photo catalysts for dye degradation.

Linear portion of last curve extrapolated to *x*-axis to find the intercept (band gap). Low Eg (1.9–2.04 eV) confirmed good absorbance in Vis. Region indicated increase density of states available for electrons occupation and high photo catalytic activity^42^.

**Figure 10:**
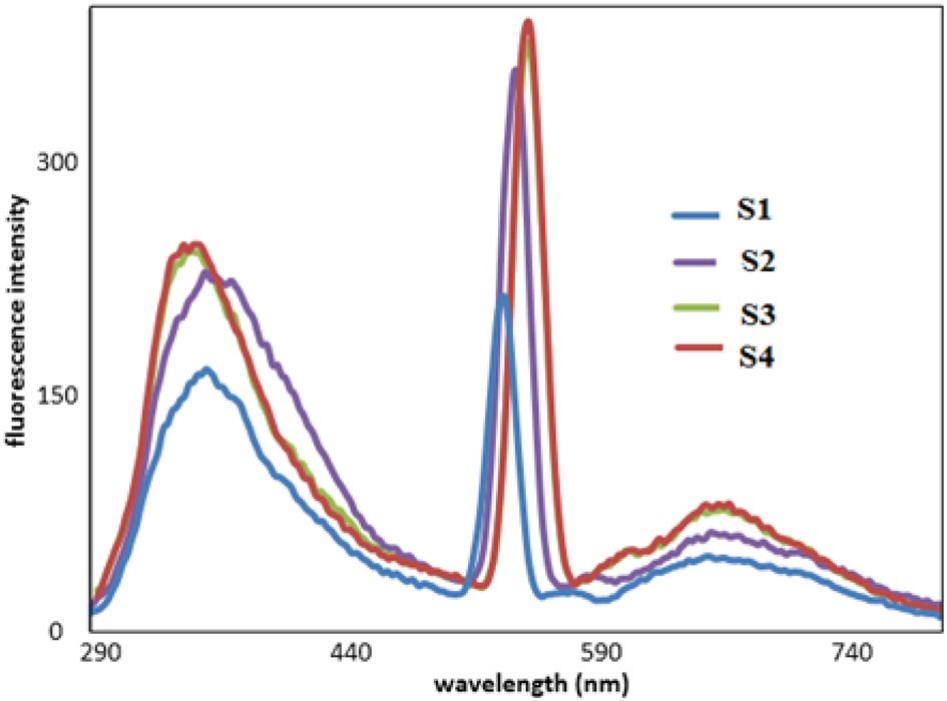
Photo lamination spectra of gelatin composites.

Figure 10 shows the nonlinear photo luminesce (PL) activity of gelatin composites on excitation at wavelength 340 nm. Optical activity: at 325–380 nm is attributed to charge transfer from gelatin ligand to Co(II) ion^44^.

Gelatin composites had large values of molar extension coefficients and long wavelength emission, Table 5.

**Table 5:** Molar extinction coefficients (ε) corresponding to wavelength of absorption and emission.

Sample No	*λ*_max abs_	*ϵ* (M^−1^ cm^−1^)*10^5^	*λ*_emission_ (excited at λ_max_._abs_.)
1	320	1.42	667
2	380	1.49	670
3	380	1.73	664
4	370	1.87	674

Sample 4 contained 20 ppm AgNPs (improved optical properties) showed the best optical activity. This composite sample showed intense absorption in Vis. region of electromagnetic radiation.

Catalytic efficiency reached 95% within 20 min. Cobalt dopant inhibited electron–hole recombination, decreasing Eg and improved photocatalytic properties, increased delocalization of electron density, and enlarged surface area^23,44^.

**Figure 11:**
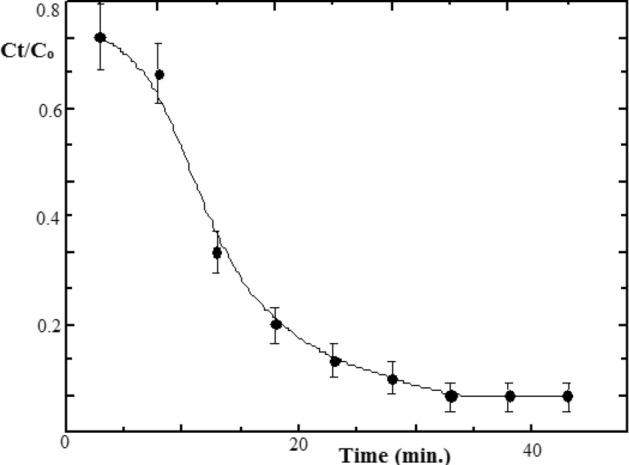
Variation of Ct/Co in photodegradation AR8 dye on the composite hydrogel.

For 300 ppm AR8 dye completely degraded into colorless solution after 90 min. contact time. Kinetics first-order model fitted photodegradation, *R*^2^ = 0.9703. The ratio of residual concentration (Ct) to initial concentration (Co) decreases rapidly with irradiation time, Fig. 11.

Gelatin composite efficiently photo degrades AR8 dye without self-splitting because of inherent attractive forces retarding solubility. Hydrogel is an efficient photocatalyst in Vis. region light-driven photodegradation of AR8 with higher %Re than conventional La photocatalyst for other pollutants^19^.

**Figure 12:**
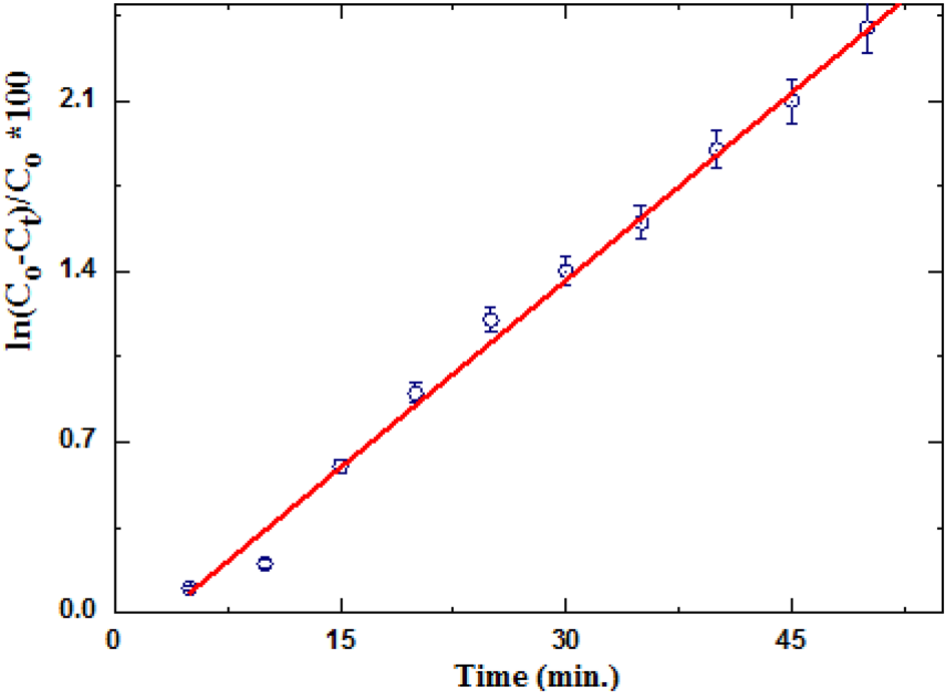
Linear fitting of photodegradation data to pseudo first-order kinetic.

Figure 12 shows a first-order plot for photodegradation data to a pseudo first-order kinetic model.

Good straight line slope (correlation coefficient R^2^) indicating that photodegradation of AR8 dye on gelatin composite followed pseudo-first order kinetically. Amount degradation unfitted to zero-order and second-order kinetic equations (R^2^ less than 0.95).

The reactant AR8 dye exists in too small concentration relative to the concentration of gelatin composite photocatalyst. The slope of the straight line equals rate constant k_1_ 5.13*10^−2^ min^−1^. The good straight line indicated degradation rates of AR8 dye increased with increasing irradiation time with dye decolonization more rapidly than La-perovskite photocatalyst for MB dye^42^.

Gelatin composite reused after immersion on 1.0 M NaOH solutions. Adsorbed hydroxyl removed dye molecules from active sites. Fig. proved hydrogel had 60% for 5th consecutive photo degradation. Biocompatible PMMA increased mechanical strength. Light stability: device tested under illumination without elevated temperatures. Heat stability: device tested without illumination at high temp (T). Shelf stability: without illumination at room T. Atmosphere humidity of stability tests not indicated. Light soaking tests (continuous 1-sun illumination) damp-heat stressors (85 °C, 85% relative humidity (RH)): Composites 90% initial wt. after 60 h in ambient atmosphere (20–30% RH) under 0.7-sun illumination. High wt.% PMMA 95% initial wt. photo stability: composite kept 100% wt. in dark, 96% on exposure inert atmosphere 10.488 h at 25 °C, illuminated at 1 sun in N_2_ at 25 °C for 100 h, wt. drop to 90% due heterogeneous interfacial layers. Lifetimes: composite retain 91 wt. % after one year aging. Phase stability: SEM micrograph, Figure after Vis. light illumination showed no phase segregation. There is no lattice Instabilities induced by strain.

### Degradation mechanism

Composite photocatalyst in an aerated aqueous solution contains AR8 irradiated under Vis. photon energy (hν), *λ*_max_. 600 nm → electron (e^−^)-positive hole (h^+^) pairs adsorbed on catalyst surface giving OH radical. Excited electron in the conduction band (CB) of gelatin composite affects adsorbed oxygen molecules from water at composite/solution interface giving superoxide radical O_2_^**.**−^. Doping gelatin by Co(II) ion created structural defects and holes giving active oxygen species (O_2_^−^, O^−^). Holes decrease Fermi level causing CB-VB characteristic with electron–hole pair.

h^+^ in VB of catalyst react with O_2_^**.**−^ generating OH^**.**^ Oxidize AR8 dye^46^.

14 $${\text{O}}_{2}^{ - } + {\text{ H}}^{ + } \to {\text{ HOO}}$$

15 $${\text{HOO}}^{.} + {\text{ e}}^{ - } \to {\text{ HO}}_{{2}}^{ - }$$

16 $${\text{HOO}}^{ - } + {\text{ H}}^{ + } \to {\text{ 2H}}_{{2}} {\text{O}}_{{2}} \left( {{\text{H}}_{{2}} {\text{O}}_{{2}} = {\text{ 2OH}}^{ - } } \right)$$

17 $${\text{H}}_{{2}} {\text{O}}_{{2}} + {\text{ e}}^{ - }_{{({\text{CB}})}} \to ^{ - } {\text{OH }} + {\text{OH}}$$

18 $${\text{H}}_{{2}} {\text{O}}_{{2}} + {\text{ h}}\nu \, \to { 2} {\text{OH}}$$

19 $${\text{AR8 }} + {\text{ OH}}^{ \bullet } \left( {{\text{Radical}}} \right) \, \to {\text{ CO}}_{{2}} + {\text{ H}}_{{2}} {\text{O }} + {\text{NH}}_{{4}} + \, + {\text{ NO}}_{{3}}^{ - } + {\text{ SO}}_{{4}}^{{ - {2}}}$$

Optically active composites are with high carrier dynamics (e-hole) separation enhanced photo degradation.

**Figure 13:**
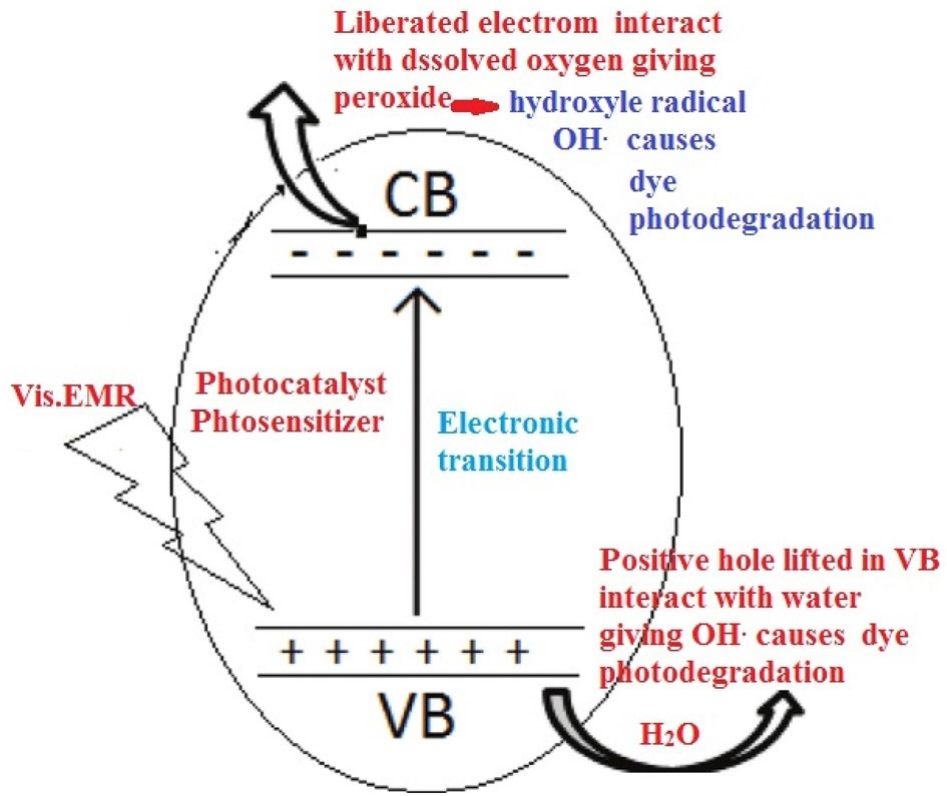
The degradation mechanism.

This mechanism could be schematically represented as following image (Fig. 13).

Holes generated in VB of catalyst oxidize dye and react with super-oxides radical, generating OH^**.**^ radicals degraded dye via one-electron redox reaction forming CO_2_, sulphate, nitrate, and H_2_O. Nitrogen compounds NH_4_^+^, and NO_3_^−^ recovered by using porous clay alumina silicate adsorbent to be used in agricultural fertilizers.

Photocatalytic activity of AgNPs on gelatin composite exceeded that of removal methylene blue (MB) over nano sized La based photocatalyst under irradiation by Vis. Light (catalytic efficiency 69% in 100 min. illumination time)^47^.

AR8 extensively polluted wastewater from textile industries and its degradation has not been reported by photocatalysts such as metal oxides and semiconductors. Efficient semiconductor catalysts with low Eg absorb in Vis. light irradiation at ambient temperature and pressure^42^. Insoluble thermally stable gelatin composite is low cos. non-toxic and easily prepared by facile low cost methods.

Electrical properties confirmed photocatalytic properties. The rough surface of the gelatin composite (sample 4) has many cavities with variable particle sizes and shapes that improve catalytic activity, Table 6.

**Table 6:** % degradation of AR8 dye in dark and light conditions.

Removal efficiency (%Re)	0	20	40	60	80	100
Dye removal in the dark by adsorption	0.03	0.6	1.05	2	6.5	6.3
Dye removal by photodegradation in Vis. light	57	69.09	76.10	83.2	86.2	96.7

Dopants CoCl_2_ and AgNPs improved photocatalytic activity by enhancing the hole-doping level and decreasing Eg. Large surface area composite enhanced photodegradation of dye. All constituents of gelatin composite are safe and have no toxicity on ecosystem^48^, Fig. 12.

Detected ions (Co (II) and Ag(I) leaching from gelatin composite during photo degradation below undeletable limitsAR8 dye ensure safety and environmental compatibility. No ion replenishment needed. Regeneration of photo catalysts required activity over prolonged periods for ensuring economic and environmental sustainability. Regeneration restoring active sites and structural integrity after fouling or consumption during photo catalysis via: washing photo catalyst with distilled water to remove adsorbed dye; Photo activation by light, simply exposing them to UV or Vis. light depending on photo catalyst’s activation range) help breaking down adsorbed pollutants, essentially self-cleaning active sites. In some cases, a mild chemical treatment dissolve any strongly adsorbed dye molecules. This could involve mild acids, bases, or oxidizing agents that do not damage the photo catalyst itself. Annealing at 60 °C in vacuum oven 1.0 h regenerate photo catalyst by decomposing adsorbed dye organic compounds. Ultrasonic waves dislodge dye molecules from active sides, cleaned with 50 mL 1.0 M NaOH. High pH causes desorption of dye molecules. Regeneration by 4.0 M NaCl increases ionic strength by factor 0.4 causing desorption of dye molecules. Each regeneration process must be tailored to specific photo catalyst and its application, considering factors such as contaminants nature of the, hydrogel composition, and economics of regeneration process. Regeneration frequency and the number of effective regeneration cycles a photo catalyst can endure are critical factors that determine the lifecycle and cost-effectiveness of the photo catalyst.

### Photodynamic activity of gelatin composites

Gelatin composite showed good optical activity: Absorption bands at 299–401 nm assigned to intra ligand metal charge transfer transition. Bands at 439–558 nm due to magnetic Oh geometry ((*µ*_eff_. 4.11 B.M).

Optically active photosensitizer (S) molecules absorb and are excited by Vis. light photon (h $\upsilon$) energy. Excited S*molecule rapidly interact with inert triplet ^3^O_2_ molecular oxygen in water producing reactive free radicals reactive oxygen species (ROS): singlet oxygen, hydroxyl radicals (OH), and superoxide (O_2_^−^) ions through charge transfer. ROS radicals contribute to oxidative damage of AR8 dye. Singlet oxygen ^1^O_2_ species rapidly attack organic dye molecules. Energetic ^1^O_2_ is very short-lived and rapidly relaxes to ^3^O_2_ after dye oxidation.

**Figure 14:**
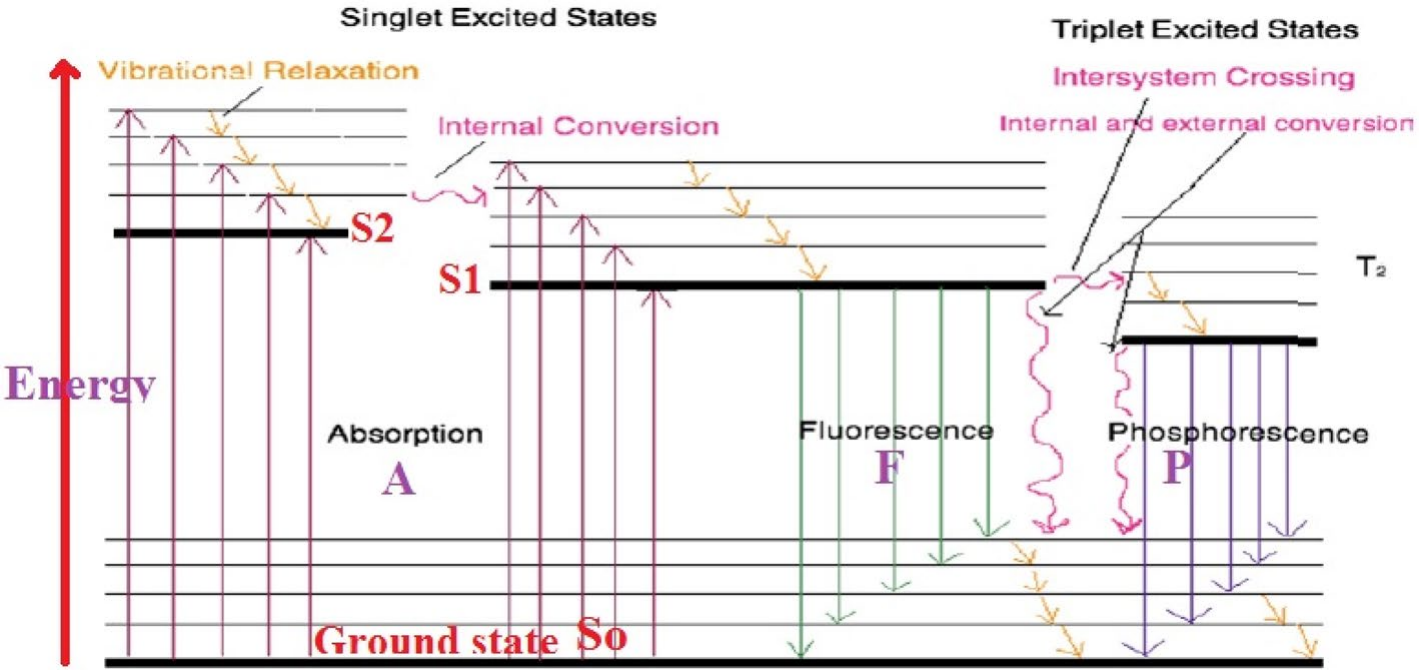
Jablonski diagram for absorption, fluorescence, and phosphorescence^49^.

S* had: efficient ISC and high T-state quantum yield (Φ_T_) and long τ allow interact with the oxygen of water.https://en.wikipedia.org/wiki/Photodynamic_therapy—cite_note-:0–5 An electron in an S moleculehttps://en.wikipedia.org/wiki/Electron is excited to a higher-energy orbital, elevating the chromophore from So into short-lived, electronically excited singlet state (Sn). Chromophore* loses energy by rapid decay through vibrational and rotational sub-levels in Sn via internal conversion (IC) to populate S1 before rapid relaxation to So. Radiative fluorescence (F) decay (S1 → So), lifetimes (τ_F_.10^−9^–10^−6^ s.) spin allowed transitions S → S or T → T conserve spin multiplicity of the electron. S1 undergoes spin inversion and populate lower-energy first excited triplet state (T1) via (ISC) spin-inversion forbidden transition followed by second spin-forbidden to depopulate excited triplet state (T1) by decaying So (phosphorescence (P) (T1 → So). (τ_P_ 0.001–1 s.) Longer than τ_F_^23^. Interaction excited T-S* with So (^3^O_2_); spin allowed transition-excited state photosensitizer: (3 S* → 1P S*, ^3^O2 → ^1^O_2_)^49^. Photocatalytic activity of excited molecule depends on τ_T_ and Φ_T_ of S*control photodynamic activity^50^, Fig. 14.

Transition metal Co(II) ion had high quantum yield (Φ_T_) and nano sec. τ_T_ showed no self-quenching of light photons before conversion ^3^O_2_ into ^1^O_2_^50^. High thermal stability of molecular structure. The dark red color confirmed that optically active chromophores interact with light photons^50^.

**Figure 15:**
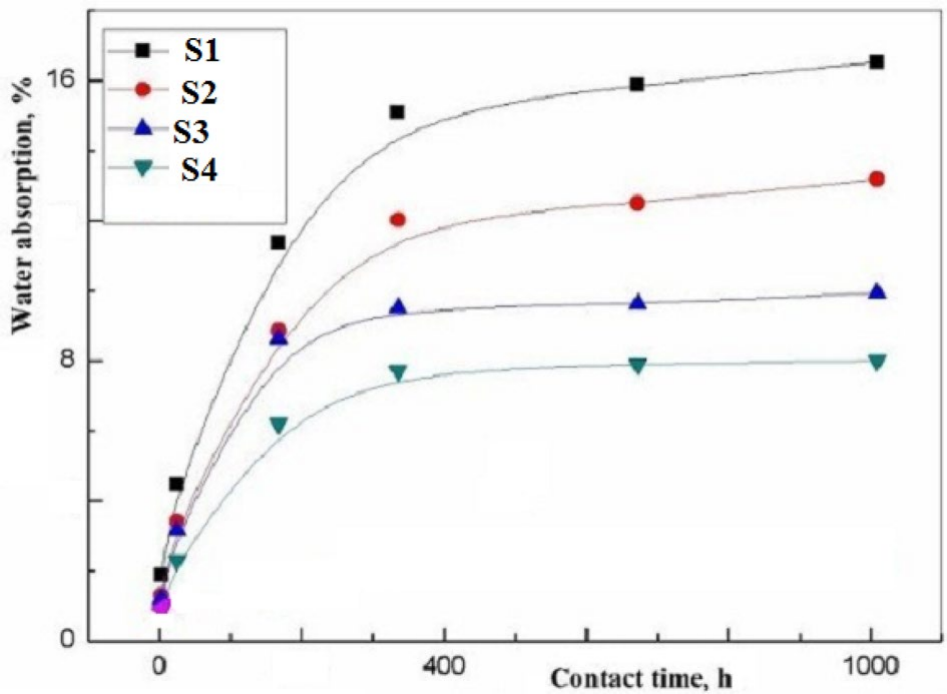
The swelling.

Dimensional stability explained improved performance of hydrophilic composites (water absorption and thickness swelling) by aqueous dye solution is essential event for photo catalysis in aqueous solutions, Fig. 15. Water easily penetrates polymer chain of hydrogel. Doping by AgNPs and CoCl_2_ decreased swelling by restriction chains movement.

### Electrical properties of gelatin composites

**Figure 16:**
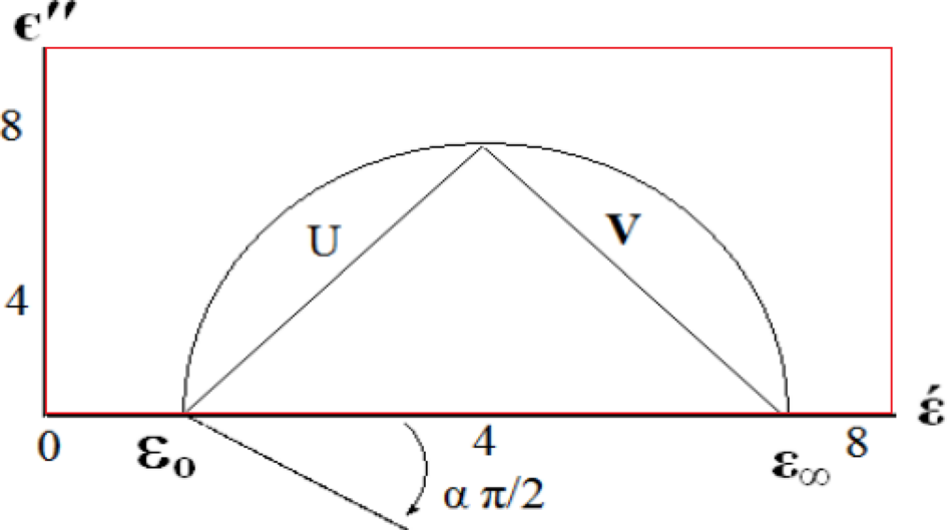
Representation variation dielectric constant with frequency.

Total conductivity σ_tot_ and dielectric parameters εˋ, εˋˋ calculated by using data of impedance (Z), capacitance (C), resistance (R), and phase angle (ϕ) at any frequency Fig. 16 illustrates dielectric properties of solid-state sample.

ε_o_ static permittivity at DC, ε_∞_ optical permittivity for very high frequencies of light oscillators. Determine distribution parameter (α) macroscopic relaxation time τ_o_, molecular relaxation timeτ, τ_o_ using relation:

20 $${\text{U}}/{\text{V }} = \, ({\text{wt}}_{{\text{o}}} )^{{{1} - {\text{a}}}}$$

where, U distance between ε_o_, observed point, V distance between that point and ε_∞_:

21 $${\text{Angular frequency}},{\text{w}} = {\text{ 2pn}}$$

where α: 0–1. Extent distribution τ increases with increasingα, τ_o_ and decreases in heating.

22 $${\text{Temperature affect}}:{\text{t}} = {\text{t}}_{{\text{o}}} {\text{exp }}\left( {{\text{E}}_{{\text{o}}} /{\text{KT}}} \right)$$

Constant τ_o_ characterizes relaxation time (single oscillation of dipole in a potential well, E_o_ free energy of activation for dipole relaxation, τ: average most probable spread relaxation time.

σ_tot_ constant at low frequency range for all samples and obeys a power relation at high-frequency range^51^:

23 $${\rm {\sigma }}_{{{\rm {tot}}}} = {\rm {\sigma }}_{{{\rm {dc}}}} \; + \;{\rm {\sigma }}^{\prime } \left( {\rm {\omega }} \right)$$

where σ_dc_ conductivity independent on frequency (extrapolation σ_tot_ at ω = 0) σˋ(ω) is Ac conductivity. Frequency dependence fitted using a power low:

24 $$\upsigma^{\prime } \left( \upomega \right)\; = \;{\rm {A}}\;\upomega^{S}$$

25 $${\rm {Dielectric}}\;{\rm {constant}}\;{\rm {\varepsilon }}^{\prime } \; = \;\frac{t}{A}.\frac{C}{{{\rm {\varepsilon }}_{o} }}$$

where C, t, and A are capacitance, thickness, and cross-sectional area of the sample respectively and ε_o_ 8.85 × 10^−12^ F/m permittivity of free space.

**Figure 17:**
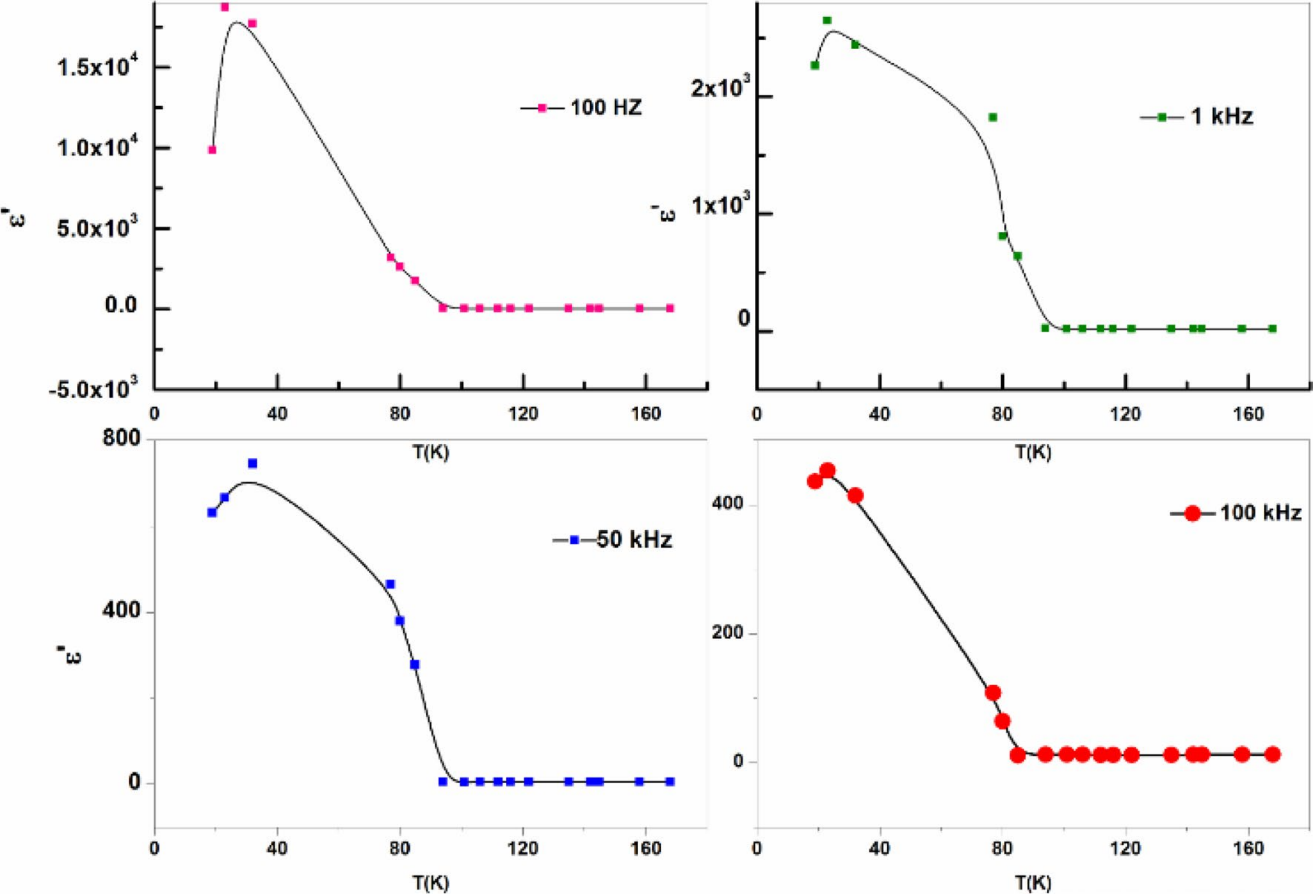
Variationε’ with frequency.

Figure 17 illustrates plot εˋ against frequency at different temperatures. Decease εˋ with increasing frequency reflects dielectric properties, ions motions, and polarity of composite. Ions rotate around their negative sites at short-distance transport (hop out of sites with low free energy barriers and pile up at sites with high free-energy barriers (ΔG activation impeding ions diffusion that vary from site to site gives different ionic motions). In electric field ion motions contribute to dielectric response cause AC conductivity. Variation dielectric properties with frequency indicating polarity. Due to dipole polarization, when AC frequency increases, ε decrease at high frequency, dielectric behavior represented in Fig. 17.

**Figure 18:**
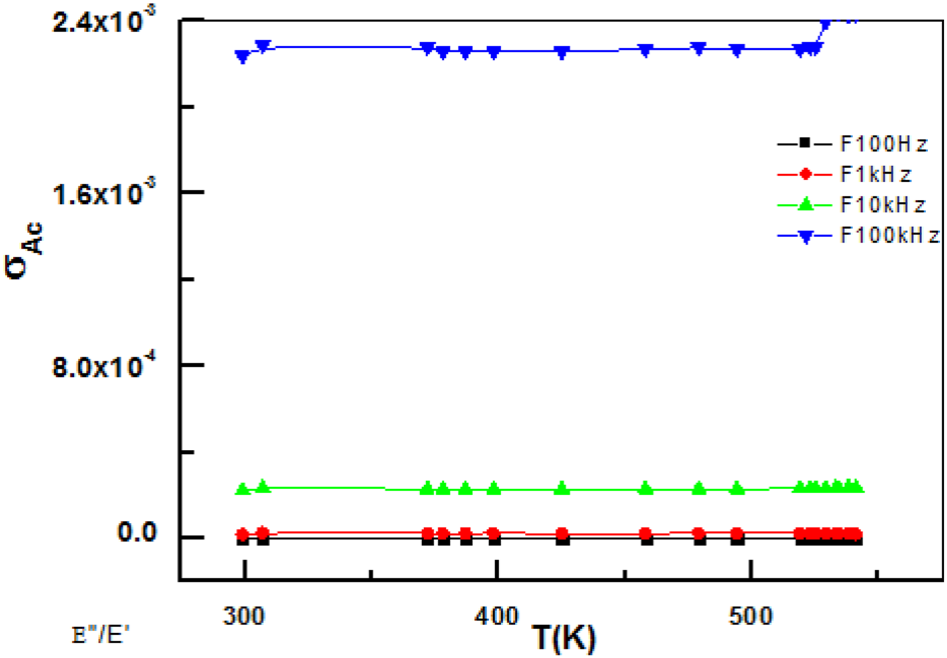
Dependence of AC conductivity on temperature and frequency.

Dielectric and electrical parameters, depend on frequency, σ (0- 26*0^−6^S/m. σ_max_ 10.13*10^6^–26.04*10^6^ Hz^51^. Dielectric constant ε’ decreased on increasing frequency due to dipole polarization and semi-conducting features on hopping mechanism.

log σ_tot_ (ω) against frequency F, Hz at room temperature showed AC conductivity increases on increasing frequency due to impedance decrease, Fig. 18.

**Figure 19:**
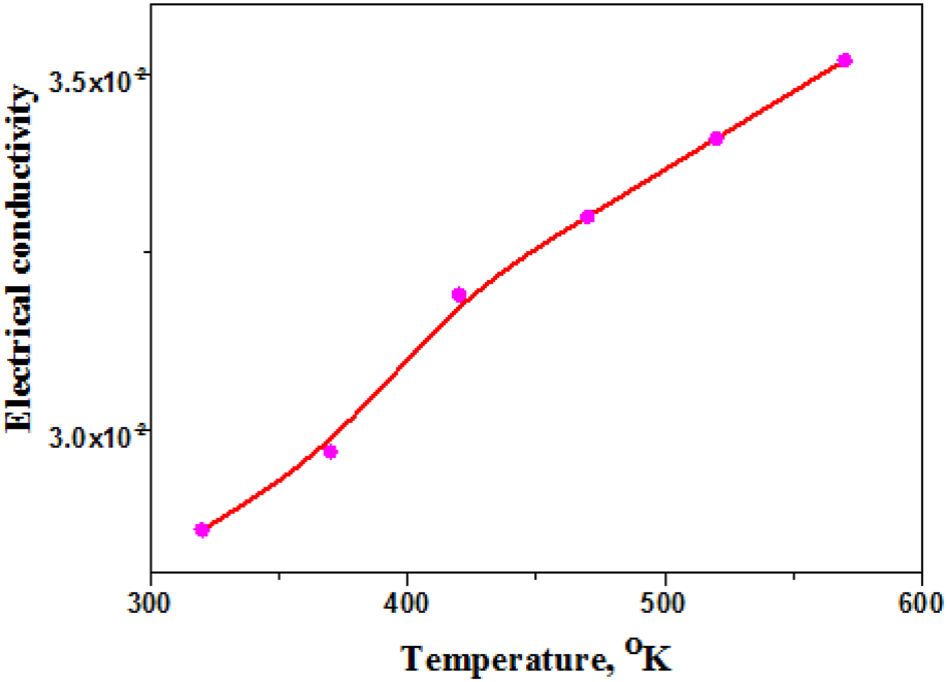
Total (AC + DC conductivity of hydrogel sample 4.

Good electrical conductivity and magnetic properties (effective magnetic moment 4.11 B.M) improved dye degradation into simple inorganic anion^51^. Variation AC conductivity with temperature, Fig. 19 indicated semiconducting properties and dielectric permittivity properties of metallic gelatin composite showed semiconducting behavior^52^.

AgNPs enhanced AC electrical conductivity 2.3 $\times {10}^{4}$ Ohm.cm^−1^ due to high electron density. This behavior explored that gelatin composites are semiconductors that have empty CB and full valence Band (VB) in electronic structure.

## Conclusion

This multidisciplinary study touching materials science, chemical engineering, setting a precedent for future interdisciplinary research in photo catalysis Explored different composites, investigation long-term stability& reusability. New red colored optically active (absorb in Vis. light, 650 nm) thermally stable photocatalysts photo catalyst prepared from sustainable gelatin polymer. Doping by CoCl_2_, AgNPs. Water-insoluble gelatin composites are for degradation toxic AR8. Defects created byCoCl_2_, AgNPs increased Eg of gelatin composites from (1.82 to 1.95) indicating interfacial charge separation. Low Eg enabled high catalytic activity in dye photo degradation. 95%Re of 500 ppm dye at pH 1.0 in 30 min., using 0.20 g L^−1^ dose photocatalyst. Dye removal mechanism is mainly photo degradation mechanism by high rate constant k_1_ 5.13 × 10^−2^ min.^−1^ Magnetic properties improved dye degradation.

Low values of Eg (ensured high absorption Vis. light ) within range of photo catysis. photo catalytic activity improved by increasing by doping and amorphous structure. Illumination 1 h revealed efficient photo catalytic activity (0.20 gL-1 dose) 95 Re%, rate constant k11.582 × 10^−2^ min^−1^. Gelatin composites are promising photo catalysts for degradation AR8 dye.

## Data Availability

All data and materials of this study are available in the manuscript and supplementary information.
